# Impact of Environmental Injustice on Children’s Health—Interaction between Air Pollution and Socioeconomic Status

**DOI:** 10.3390/ijerph18020795

**Published:** 2021-01-19

**Authors:** Sahana Mathiarasan, Anke Hüls

**Affiliations:** 1 Department of Epidemiology, Rollins School of Public Health, Emory University, Atlanta, GA 30322, USA; sahana.mathiarasan@emory.edu; 2 Gangarosa Department of Environmental Health, Rollins School of Public Health, Emory University, Atlanta, GA 30322, USA

**Keywords:** environmental injustice, children, respiratory health, cognitive development, air pollution, socioeconomic status

## Abstract

Air pollution disproportionately affects marginalized populations of lower socioeconomic status. There is little literature on how socioeconomic status affects the risk of exposure to air pollution and associated health outcomes, particularly for children’s health. The objective of this article was to review the existing literature on air pollution and children’s health and discern how socioeconomic status affects this association. The concept of environmental injustice recognizes how underserved communities often suffer from higher air pollution concentrations in addition to other underlying risk factors for impaired health. This exposure then exerts larger effects on their health than it does in the average population, affecting the whole body, including the lungs and the brain. Children, whose organs and mind are still developing and who do not have the means of protecting themselves or creating change, are the most vulnerable to the detrimental effects of air pollution and environmental injustice. The adverse health effects of air pollution and environmental injustice can harm children well into adulthood and may even have transgenerational effects. There is an urgent need for action in order to ensure the health and safety of future generations, as social disparities are continuously increasing, due to social discrimination and climate change.

## 1. Introduction

Air pollution is the increase of pollutant substances in the atmosphere due to human activity, such as industrialization, urbanization, and traffic pollution, as well as natural sources, like wild fires or volcanic eruptions [1,2,3]. While high-income countries, such as countries in Western Europe and the USA, see a decrease of air pollution concentrations, air pollution concentrations are increasing in low- to middle-income countries, such as India and China [4]. While traditional household pollutants have decreased, mass industry and vehicle related pollution have increased, especially in low- to middle-income countries [2]. As low- to middle-income countries continue to rapidly industrialize, air pollution concentrations increase [2]. More people in these countries are now able to afford vehicles, which increases traffic related air pollution [2]. Air pollution causes up to seven-million premature deaths yearly [5]. These deaths are disproportionately in areas of lower socioeconomic status in low- to middle-income countries [2]. Low air quality is associated with a plethora of negative health outcomes, including respiratory [6] and neuropsychological [7,8] health problems.

In addition, there are also substantial differences in the vulnerability to air pollution within countries. Underserved communities often suffer from high concentrations of air pollution, as well as social risk factors and racism and poverty, which is summarized under the concept of environmental injustice. Environmental injustice recognizes how air pollution can disproportionately affect marginalized populations, such as communities with lower socioeconomic status [9], which is a person’s combined economic and social standings. In high-income countries, communities with low socioeconomic status are much more likely to have a higher chronic disease burden than communities with high socioeconomic status [10]. In the United States, social inequalities are generally characterized as “disparities” and attention has focused primarily on racial and ethnic differences and gender differences [11]. Elsewhere, the focus has been largely on differences between poor and wealthy countries and, within countries, across social groups that are characterized by income, education, occupation, or immigrant status [12].

Low socioeconomic status affects many aspects of a person’s life and it is often accompanied by risk factors, such as malnutrition and lack of access to healthcare. Factors, such as lower socioeconomic status as well as discrimination, can also cause social and psychological stress, which makes the body more susceptible to infections and diseases [13]. Stress also increases the risk of developing negative health outcomes due to exposure to air pollution. Individuals from communities with lower socioeconomic status often experience higher levels of air pollution, which lead to detrimental health problems [14]. Examples include excess indoor air pollution concentrations from smoke or cooking fuels and increased exposure to outdoor air pollution concentrations due to housing near factories and major roads. Socioeconomic status and its effects on health may also vary due to the type of community that a person is in. While air pollution concentrations tend to be higher in urban areas, the individual cumulative exposure of children from rural areas is sometimes even higher, depending on how much time they spend outside. Consequently, one study found that rural areas are associated with higher risks of mortality due to air pollution when compared to urban areas [15]. Health disparities as well as other underlying risk factors that stem from the lack of health-related resources and access to them may also lead to the increased negative risks of air pollution.

When looking at children’s health, social determinants, such as socioeconomic status, must be taken into account to better help the most vulnerable population. Children inhale disproportionately more air pollution than adults in the same environment due to their lower body weights [16]. Children do not have the means of protecting themselves or creating change; however, their organs and brain are in the process of development, which makes them more susceptible to the harmful effects of environmental pollutants [17]. Children’s bodies also take more time to process and excrete toxic materials, meaning that pollutants stay longer in their systems due to a less developed metabolic system [9].

While there are many articles that link air pollution to negative health outcomes, little research has been done that takes into account socioeconomic status and how it directly affects the association between air pollution and children’s health. This review article explores how children’s socioeconomic status can increase their exposure to air pollution and affect their risk of respiratory and cognitive health effects. The article also delves deeper into the differences between different indoor and outdoor air pollutants and how children of lower socioeconomic status are often exposed to both types of air pollution.

## 2. Impact of Environmental Injustice on Children’s Health

### 2.1. Socioeconomic Status

Socioeconomic status affects the amount and quality of care that a person can receive [18]. The lack of access to healthcare is a large problem for people of lower socioeconomic status. They are often subject to longer wait times in hospitals, which can be very detrimental, as that is time taken away from working and making money [19]. This can deter people from seeking medical attention for health problems. Socioeconomic status also affects access to treatment and medicine and their ability to cover medical expenses. Implicit bias against people of lower socioeconomic status can also be a barrier to adequate care [20]. In low income countries, children oftentimes are forced into child labor in order to help provide for their families [21], which can result in various health problems due to dangerous working conditions.

Socioeconomic status can also affect other parts of a child’s life, which can lead to morbidities and increase the vulnerability to air pollution-related detrimental health effects. Nutrition is one of the factors that is affected by lower socioeconomic status. Malnutrition during early childhood is associated with an increased risk of disease as well as physical and cognitive developmental effects [22]. Food insecurity, as well as other issues that contribute to lower quality of life, can also lead to negative effects in a child’s academic performance [23]. Lower levels of education make it difficult for children to leave poverty. Other factors, such as social discrimination, can cause physical as well as emotional and mental damage in children [24]. These additional problems that children face due to lower socioeconomic status can increase their risk of developing negative health outcomes due to air pollution.

### 2.2. Air Pollution

**Figure 1:**
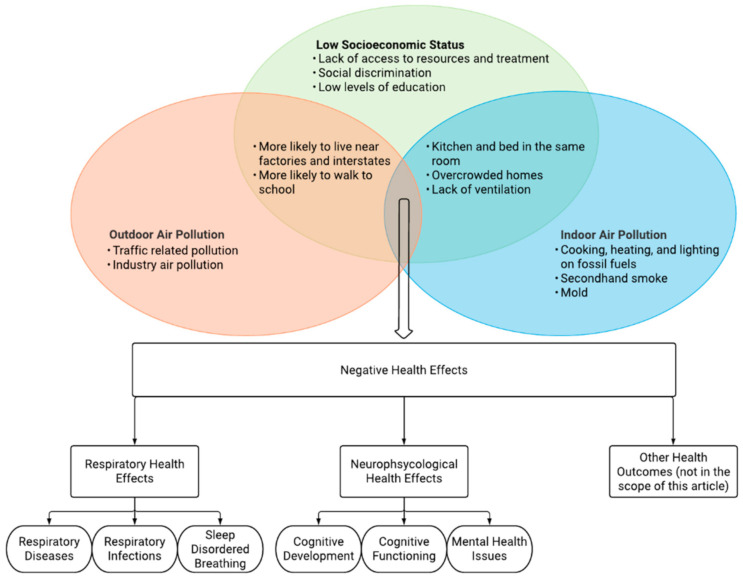
Environmental injustice. The detrimental intersection of outdoor and indoor air pollution with socioeconomic status and its consequences for children’s health.

Air pollution provides one of the highest environmental health risks for underserved communities [25]. The intersection of outdoor and indoor air pollution with socioeconomic status can result in many adverse health outcomes (Figure 1). Indoor and outdoor air pollution can both result in similar physical health problems, as they both affect the body through similar mechanisms. Indoor air pollution is still a major problem for child health, particularly in low to middle income countries [26]. The respiratory and neuropsychological effects of indoor and outdoor air pollution may be similar, but their unique ties with socioeconomic status can result in different morbidities as well as social challenges. The amount of indoor and outdoor air pollution that a child is exposed to can also increase with lower socioeconomic status.

#### 2.2.1. Outdoor Air Pollution

Outdoor air pollution mainly comes from traffic and industry related pollution, such as power plants [27]. Outdoor air pollution is mainly associated with negative respiratory health outcomes [28]. Specific air pollutants were found to be linked to sleep disordered breathing in children, such as wheezing and snoring [29]. Inadequate sleep is associated with many other health problems, such as heart disease [30] and obesity [31]. An increased risk of developing asthma has also been linked to outdoor air pollution and air pollution may also exacerbate existing asthmatic conditions through oxidative stress and inflammation in air pathways [27]. More recent research has shown that outdoor air pollution can also have detrimental effects on the developing brain. The brain develops at a rapid pace during childhood and, therefore, is more susceptible to environmental factors. Young children are unprotected from environmental factors, such as air pollution, which can induce neurotoxicity and significantly alter the brain and cause serious health outcomes [32]. Outdoor air pollution has been associated with a child’s academic performance [33], which can further continue the cycle of income inequality, as higher education allows for people to get jobs with higher salary. Furthermore, traffic-related air pollution is associated with structural alterations in the brain, such as reduced regional grey matter and cortical thickness [34]. Children from schools in highly polluted environments had a smaller growth in cognitive development than children from the schools in lowly polluted areas. Air pollution can also inhibit a child’s working memory [35], which can negatively impact their academic performance. Air pollution might also affect children’s mental health. Exposure to air pollution during infancy [36] and prenatal stages [37] has been associated with autism spectrum disorder (ASD) and long-term exposure to air pollution during childhood has been associated with an increased risk of developing attention-deficit/hyperactivity disorder (ADHD) [38,39] and depression [8].

Children of lower socioeconomic status are disproportionately exposed to higher rates of outdoor air pollution. They are more likely to walk to school or use public transport, which makes them more vulnerable to environmental factors [40]. They are also more likely to live in communities that are close to factories or major roads that may be polluting the surrounding area and less likely to have resources like air conditioning to filter outdoor air pollution [41]. The type of community that a child lives in can also affect how much air pollution they are exposed to. While urban areas may have higher concentrations of air pollution, children in rural areas often spend more time outside compared to children in urban areas [42], which could increase the cumulative amount of outdoor air pollution that they are exposed to. Moreover, a lack of access to care and inadequate care makes children of lower socioeconomic status more vulnerable to the detrimental effects of environmental factors, such as air pollution. In times of climate change, these disparities will continue to increase. Children of lower socioeconomic status are less likely to have the opportunity to relocate when natural disasters occur. They are also more likely to live in houses that are more easily affected by natural disasters. As air quality continues to worsen due to the consequences of global warming [5], children of lower socioeconomic status will face worse health outcomes from increased exposure to air pollution as well as other environmental pollutants.

#### 2.2.2. Indoor Air Pollution

Indoor air pollution can come from sources, such as fuels used for cooking, heating, and lighting, and secondhand smoke [43]. Other living aspects, such as poor ventilation, can also increase the risk of exposure to indoor air pollution. Indoor air pollution is associated with detrimental respiratory health effects in children, such as lower respiratory tract illness and wheezing [44]. Children living in houses with no separate kitchen or in houses using polluting fuels as opposed to clean fuels were found to have an increased risk of pneumonia [45]. Subnormal lung function was found in children that were exposed to air pollution in the household due to solid fuels that were used in the home [46]. Childhood asthma and acute lower respiratory infections are also linked to indoor air pollution as well as poor ventilation in houses [43,47]. Similar to outdoor air pollution, indoor air pollution exposure has also been linked to cognitive development. Children attending schools with higher concentrations of indoor air pollution experienced substantially smaller growth in all cognitive measurements [35]. This can affect both their academic experience and performance.

Children in households of lower socioeconomic status are more likely to not have separate living and kitchen spaces, which increases their exposure to indoor air pollutants. Cooking fuels that create indoor air pollution are more likely to be used in households of lower socioeconomic status, as access to resources, like gas and electricity, is limited. Children in households of lower socioeconomic status are also more likely to live with more people in a smaller space and have less ventilation in their homes [43,45].

## 3. Biological Mechanisms

Children of lower socioeconomic status are exposed to higher concentrations of both outdoor and indoor air pollution. As a result of this, they are more likely to face changes in biological mechanisms in response to air pollution exposure. As air pollution and social stress trigger the same biological pathways, it is likely that the effects of air pollution and stress that are caused by low socioeconomic status are similar and potentially synergistic [13,48]. Excess air pollution can result in an inflammatory response in the body. Prolonged exposure to air pollution can result in increased oxidative stress, which is the increase of free radicals in the body [48]. This can lead to the elevated production of specific proteins, called monocytes, a type of white blood cells, which create inflammation [49]. Consequently, ambient particulate matter can induce airway inflammation [50,51] and exacerbate inflammation that is related to existing diseases, such as glucose metabolism disorders [52] and asthma [53]. Air pollution can also cause neuroinflammation, which is associated with many adverse neurodegenerative results. Diseases, such as Parkinson’s and Alzheimer’s, have been linked to exposure to air pollution [54]. Chronic inflammation leads to the destruction of healthy cells which could cause DNA and tissue damage. Consequently, chronic inflammation is associated with a multitude of chronic diseases and even cancer. While there is a correlation between air pollution and inflammation, the specific underlying biological pathways require more research in order to be better understood. People of lower socioeconomic status are more likely to face excess air pollution concentrations and, therefore, are more inclined to develop inflammation, which could adversely affect their health [55].

Air pollution can also create changes in histones and chromatin, which affects DNA methylation [56]. Prenatal exposure to air pollution is also associated with mitochondrial dysfunction as well as changes to mitochondrial DNA. Damage to the mitochondria can result in many negative health outcomes, as it directly affects cell energy expenditure and processes. Exposure to air pollution is associated with differential DNA methylation levels, which can potentially lead to transcriptional and translational issues, as methylation is one of the primary methods of gene regulation. As the processes of growth and regulation are still developing during childhood, changes to their DNA can cause detrimental health effects, such as inflammation and disease [57]. Prenatal and postnatal exposure to pollution, such as smoke due to maternal smoking during and after pregnancy, has also been linked to changes in DNA methylation and it can cause lifelong health issues that can start soon after a child is born [57]. People of a lower socioeconomic status are more likely to partake in smoking [58], so the risk of children of lower socioeconomic status being exposed to secondhand smoke is higher, increasing their risks of negative health effects. Changes in DNA methylation are associated with the development of many cancers, especially lung cancer [59]. Sustained exposure to air pollution can also lower the chances of recovering from cancers [59], which is more likely for people of lower socioeconomic status, as they are less likely to be able to leave the situation that they are in [59]. More research is needed in order to fully understand the role of DNA methylation for the association between air pollution and children’s health [60].

## 4. Discussion

Air pollution disproportionately affects marginalized populations of lower socioeconomic status. Children of lower socioeconomic status are more likely to be exposed to both indoor and outdoor air pollution. Children are especially vulnerable to these pollutants, as their bodies are still developing and cannot process toxic materials as fast as adults. They are also more likely to develop chronic conditions from early exposure to pollutants. Air pollution is associated with negative respiratory health effects such as diseases, infections, and sleep disordered breathing. It can also lead to negative neuropsychological effects, such as developmental delays, structural alterations in the brain, slower working memory, and mental health issues. Air pollution can also create detrimental changes to DNA methylation in children, as well as increase oxidative stress and inflammation, leading to lifelong health problems, such as cancer and other diseases. These adverse health effects can harm children well into adulthood and they may also have transgenerational effects. Issues, such as climate change, continue to increase the risk for marginalized communities.

### 4.1. Social and Racial Discrimination

Many aspects of a child’s physical health can be affected by their socioeconomic status. In addition, low socioeconomic status can also affect a child’s emotional and mental health, as they may face discrimination which could add to the stress that they feel [61]. The lack of mental health resources could increase the chances of developing mental health issues and social and psychological stress are associated with increased oxidative stress levels in the body [62]. Proper nutrition and sleep could combat oxidative stress; however, children of lower socioeconomic status are less likely to receive proper nutrition and they can develop sleep problems due to many different factors, including air pollution.

Other social determinants, such as race, are associated with lower socioeconomic status and can affect children’s health. Socioeconomic status and race are closely tied together, and they account for the disproportionate levels of disease in populations of color and many of the disparities in access to healthcare due to racism [63]. The lack of access to healthcare as well as a lack of adequate care for people of color can create many negative health outcomes, such as higher rates of morbidity and mortality [64]. Children of lower socioeconomic status can face disadvantages, due to many other reasons and social determinants. These issues can compound to create many different detrimental health effects in their future.

### 4.2. Adulthood and Transgenerational Effects

The effects of air pollution not only affect people in childhood, but also follow them in adulthood. Even if children become less exposed to air pollution as young adults, they are still more likely to have decreased lung function and more at risk for respiratory diseases and infections due to childhood exposure [65]. Childhood environmental exposure to air pollution, such as second-hand smoking, can also lead to chronic diseases, such as emphysema [66]. Chronic diseases that are developed during childhood significantly affect people’s lives during adulthood and put them at a disadvantage. The lack of resources and adequate health care can also lead to the promotion of the cycles of poverty and oppression. With low socioeconomic status and poor health, it is very difficult for people to escape poverty. Air pollution has also been found to be linked with higher rates of child mortality [67], so children are less likely to make it to adulthood and they will be burdened with health problems if they do.

Environmental injustice does not only have a negative impact on the current generation, but also on their children and grandchildren. Low socioeconomic status can lead to a lack of education which hinders children from going into higher paying fields when they are older. This continues the cycles of poverty and oppression throughout multiple generations. There are also possible transgenerational epigenetic effects that are caused by exposure to pesticides, lead, and diesel exhaust fumes, which disproportionately affect marginalized communities. This means that further generations can also be affected by the effects of air pollution, even if they are not directly exposed to it [56]. Air pollution is associated with changes in histones and chromatin, which affects DNA methylation [56]. These changes can occur during prenatal or postnatal stages and they can be passed down to future generations. Environmental justice is needed not only for the wellbeing of children now, but also future generations.

### 4.3. Climate Change

As socioeconomic status continues to be a barrier for children of lower socioeconomic status, they will also be subject to other stressors that will disproportionately affect their health, such as climate change. Climate change could also increase the risk of disease due to changing and extreme weather patterns [68]. Changing weather patterns are associated with the increase in allergens as well as disease vectors, which is associated with the increase in prevalence of respiratory conditions and diseases, such as asthma [69]. Climate change will increase the number of changes in weather as well as natural disasters. It is also associated with the increase of phenomena such as winter inversion, which is when a layer of cold air is trapped under a layer of warm air during the winter and greatly increases the air pollution level in that region [70]. People of lower socioeconomic status are less likely to have the resources to be prepared for these events, thus increasing the number of negative outcomes that they will face [71]. They are also less likely to have the ability to move or evacuate from affected areas. A lack of access to health care will also provide underserved communities with obstacles for recovering from the health effects caused by these disasters. Marginalized populations are also less likely to be prepared for the changes in temperature that could occur from climate change [71]. Climate change will hurt underserved communities most and it is a threat to the basic necessities that they need for life. Different strategies must be applied to help the communities, especially during national disasters. Primary care providers must be well trained and informed regarding how to be adequately prepared to help these communities [72]. Combating climate change must be addressed by various governments with the aid of primary care providers [71]. Without adequate measures that help vulnerable populations, the mortality rate due to air pollution will continue to increase [5].

## 5. Conclusions/Recommendations

More research is needed in order to effectively recommend what steps must be taken to better protect marginalized communities from the effects of air pollution. Large projects, like the PINCHE project, have been funded in Europe [73,74,75]; however, these projects do not take sociocultural factors, such as socioeconomic status, into consideration. Socioeconomic status is also not the only social determinant that can affect health. Intersectional research on aspects, like gender and race, must also be done to better understand and help marginalized populations and their unique circumstances. Action must be taken through an interdisciplinary approach that combats air pollution and environmental injustice in order to ensure the health and safety of future generations.

## Data Availability

Not applicable.
